# Identification and testing of oviposition attractant chemical compounds for *Musca domestica*

**DOI:** 10.1038/srep33017

**Published:** 2016-09-26

**Authors:** Rui Tang, Feng Zhang, N’Golopé Kone, Jing-Hua Chen, Fen Zhu, Ri-Chou Han, Chao-Liang Lei, Marc Kenis, Ling-Qiao Huang, Chen-Zhu Wang

**Affiliations:** 1 MoA-CABI Joint Laboratory for Bio-safety, Institute of Plant Protection, Chinese Academy of Agricultural Sciences, 2 Yuanmingyuan West Road, Beijing 100193, P. R. China; 2 State Key Laboratory of Integrated Management of Pest Insects and Rodents, Institute of Zoology, Chinese Academy of Sciences, 1-5 Beichen West Road, Beijing 100101, P. R. China; 3 CABI East Asia, Chinese Academy of Agricultural Sciences, 12 Zhong Guancun South Street, Beijing 100081, P. R. China; 4 Institut D’Economie Rurale, CRRA-Sotuba, BP 262, Bamako, Mali; 5 Guangdong Entomological Institute, Chinese Academy of Sciences, 100 Xianlie Road C, Guangzhou 510070, P. R. China; 6 Huazhong Agricultural University, No. 1 Shizishan Street, Hongshan District, Wuhan, Hubei Province, 430070, P. R. China; 7 CABI, 2800 Delémont. Switzerland

## Abstract

Oviposition attractants for the house fly *Musca domestica* have been investigated using electrophysiological tests, behavioural assays and field tests. Volatiles were collected via head space absorption method from fermented wheat bran, fresh wheat bran, rearing substrate residue and house fly maggots. A Y-tube olfactometer assay showed that the odor of fermented wheat bran was a significant attractant for female house flies. Bioactive compounds from fermented wheat bran for house fly females were identified by electrophysiology and mass spectrophotometry and confirmed with standard chemicals. Four electrophysiologically active compounds including ethyl palmitate, ethyl linoleate, methyl linoleate, and linoleic acid were found at a proportion of 10:24:6:0.2. Functional imaging in the female antennal lobes revealed an overlapped active pattern for all chemicals. Further multiple-choice behavioural bioassays showed that these chemicals, as well as a mixture that mimicked the naturally occurring combination, increased the attractiveness of non-preferred rearing substrates of cotton and maize powder. Finally, a field demonstration test revealed that, by adding this mimic blend into a rearing substrate used to attract and breed house flies in West Africa, egg numbers laid by females were increased. These chemicals could be utilized to improve house fly production systems or considered for lure traps.

The house fly, *Musca domestica* L., is a recurrent pest in animal farms and many other human habitats because of its annoyance capacity and its ability to carry animal and human diseases^1^,^2^. House flies are particularly difficult to manage because of their ability to feed and reproduce on many types of organic matter, their short developmental cycle and their resistance to insecticides^3^. Thus, new control methods are greatly needed. On the other hand, *M. domestica* is also increasingly considered as a protein source for livestock feed, and/or as a method of waste management, and as such mass rearing systems are being developed worldwide^4^,^5^,^6^,^7^,^8^. Egg production has been identified as one of the bottlenecks of such production systems^7^,^9^. Therefore, any substance that may attract *M. domestica* and enhance oviposition would be useful, both for increasing egg production in mass rearing systems and for the development of new lures for trapping systems.

*Musca domestica* employs (*Z*)-9-tricosene^10^ as a major component of its sex pheromone. In addition, cis-9, 10-epoxytricosane, (*Z*)-14-tricosene-10-one^11^ and a complex mixture of methyl alkanes^12^,^13^ have also been reported as minor compounds which, together, comprise the sex pheromone chemical blend for this species. House fly sex pheromone is known to be involved in mating behaviours such as short-range location, sex-recognition and arrestance^14^. (*Z*)-9-tricosene has been identified from female ovaries and confirmed as an oviposition attractant for females^15^. However, oviposition is not only influenced by (*Z*)-9-tricosene, but also by the odors and microbiol composition of substrates in which females lay eggs^16^. For example, fermented wheat bran could attract female house flies to oviposit regardless of the presence of pheromone components^15^. Dimethyl trisulfide in head space absorbed volatile samples from incised rat carrion has been reported as an oviposition attractant in green bottle fly *Lucilia sericata* Meigen^17^.

Mass production systems for house fly larvae use fermented pig manure, fermented chicken manure and fermented wheat bran as substrates^6^,^7^. Fermented wheat bran is a very efficient substrate and it is considered clean, reasonably cheap and highly nutritious for fly larvae. Though volatile compounds have been identified from rearing substrates such as pig manure^18^, so far no candidate oviposition attractant chamicals have been reported from wheat bran volatiles.

In order to identify bioactive volatiles from fermented wheat bran and evaluate the potential use of these chemicals as candidate oviposition attractants for *M. domestica* females, we introduced a combination of bioassays, electrophysiological tests and field trials to screen and demonstrate their performance both in the laboratory and in the field.

## Results

### Y-tube olfactometer test

**Figure 1:**
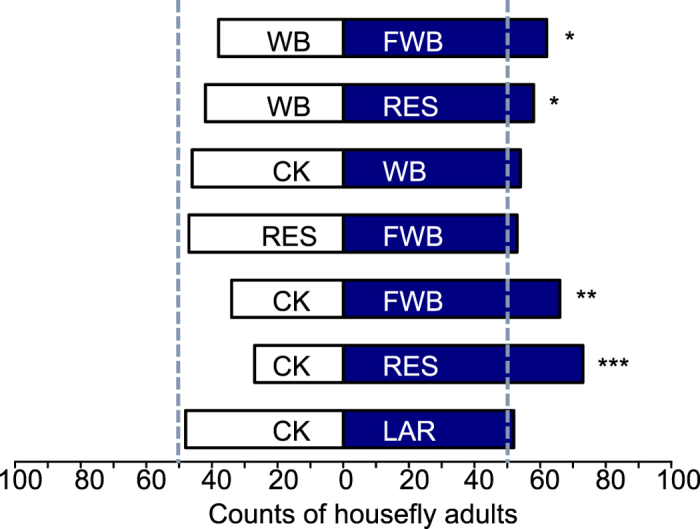
Results of two-choice tests by *Musca domestica* females in Y-olfactometers. X-axis digits indicate counts of female house flies. FWB: fermented wheat bran, WB: wheat bran, RES: residue, CK: solvent control, LAR: larvae odor; asterisks indicate significant differences between the two choices, *p < 0.05, **p < 0.01, ***p < 0.001, *Chi-square test*.

In two-choice tests, 700 out of 748 females and 182 out of 220 males have exhibited choice action. Females showed a significant preference for fermented wheat bran (*Chi-square* test, P = 0.001) and rearing residues (*Chi-square* test, P < 0.0001) over fresh wheat bran and the control (Fig. 1). No preference for larval odors was observed as compared to the control (Chi-Square = 0.16, P = 0.689). No substrate preference was observed in males (Supplementary Fig. S1).

### Bioactive compounds identification

Ten chemicals were identified in fresh wheat bran odors, 16 in larval odor, 18 in fermented wheat bran and 25 in rearing residues (Supplementary Table S1). The composition and proportion of the chemicals dramatically changed through the fermentation and feeding processes. In particular, various ester compounds were produced by the fermentation process.

**Figure 2:**
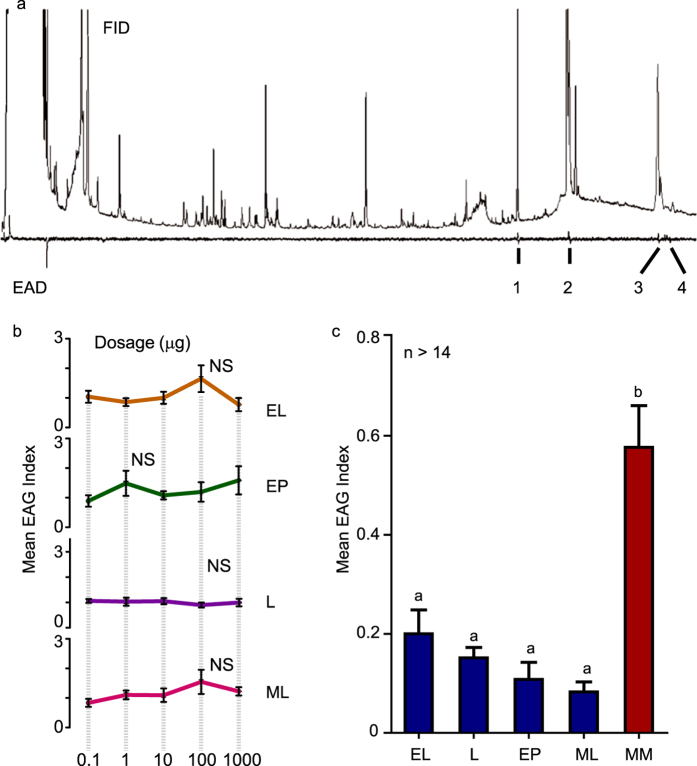
(**a**) Representative amplitude pattern of GC-EAD results for *M. domestica* female to fermented wheat bran odor blend. 1: Ethyl palmitate, 2: Ethyl linoleate, 3: Methyl linoleate, 4: Linoleic acid. (**b**) EAG responses of female antennae to four standard chemicals at five dosages. EAG Index was calculated with R_C_−(R_C−1_−R_C+1_)/2; R_C_: recorded value, R_C−1_: control value before recording, R_C+1_: control value after recording. Error bars indicate ± s.e.m. (**c**) Comparison among four standard chemicals and MIMIC odorant blends at a dose of 100 ug for female *M. domestica* adults. Lowercases indicate differences of electrophysiological response index among five treatments (EL: ethyl linoleate, EP: ethyl palmitate, L: linoleic acid, ML: methyl linoleate, MM: mimic blend; *ANOVA, Tukey HSD*, F_4, 302_ = 11.8, P < 0.0001). Error bars indicate ± s. e. m.

The electroantennogram (EAG) signal stimulated by single chemicals and confirmed by standard chemical mass spectrum are shown for fermented wheat bran in Fig. 2a. Four bioactive compounds were identified including ethyl palmitate (EP), ethyl linoleate (EL), methyl linoleate (ML) and linoleic acid (L) with a proportion of 10:24:6:0.2. The total concentration of the four chemicals was 40.63 μg/ml, which was calculated via an external standard curve developed from the EP standard solution. According to the air flow rate (1 L/min), sampling size (10 g dry weight) and sampling time (8 h), the estimated emission rate for these compounds was 8.46 ng/min · g · L. Four other chemical compounds stimulating signals in female antennae were found in the rearing residue odor blend: decane, 2-butyl-1-octanol, nonadecane and squalene (Supplementary Fig. S2). However, rearing residues are known to be neglected by females as oviposition substrates because they are not favorable for larval development^19^,^20^, and thus we did not choose these chemicals for further oviposition tests. Neither fermented wheat bran odor nor residue odor stimulated EAG responses in male antennae (Supplementary Fig. S2).

In the EAG test, no significant dose-dependent responses were found within the testing range of each chemical (Fig. 2b, *ANOVA*, EL: F_4, 42_ = 2.378, P = 0.067, EP: F_4, 41_ = 1.157, P = 0.344, L: F_4, 41_ = 0.756, P = 0.560, ML: F_4, 41_ = 2.014, P = 0.110). However, a MIMIC (MM) blend of the four chemicals at the same proportion as the extracted samples showed a significantly higher intensity in the responses of female antennae as compared to individual (EL, EP, ML, and L) chemicals (Fig. 2c, *ANOVA, Tukey HSD*, F_4, 302_ = 11.8, P < 0.0001). In contrast no difference among the five treatments was observed for male antennae responses (Supplementary Fig. S3, *ANOVA, Tukey HSD*, F_4, 61_ = 2.45, P = 0.055).

### Activation patterns in female house fly antennal lobes (AL)

**Figure 3:**
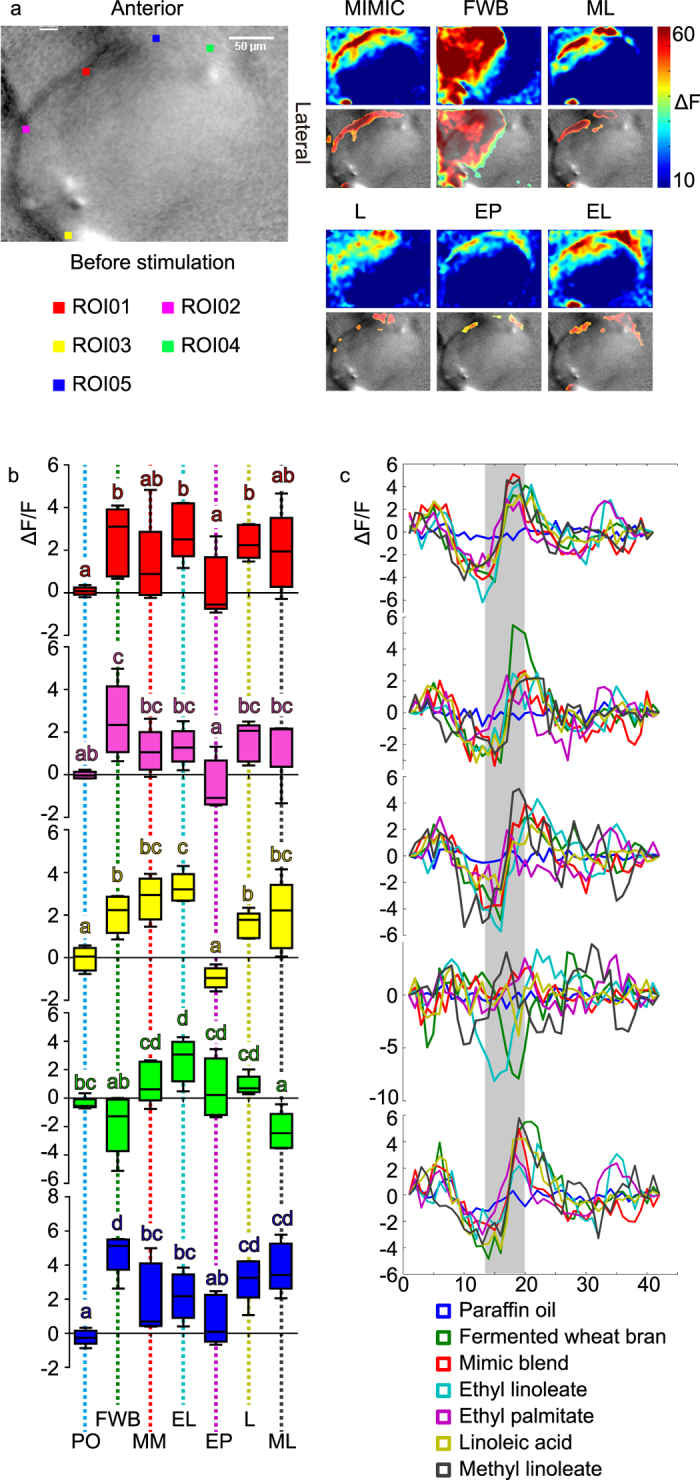
(**a**) Spatial coding patterns of antennal lobes responded to six samples in female *M. domestica* adults. Color squares indicate regions of interested (ROI). (**b**) Comparison among six tested samples and paraffin oil control in each ROI region respectively. Plots with no letter in common indicate significant differences among tested samples. Error bars indicate ± s. e. m. (**c**) Spatial-temporal pattern in each ROI for all chemical samples. Stimulation started at frame 13.

The odorant blend of fermented wheat bran (FWB) evoked the largest spatial activity pattern in female house fly antennal lobe and the area overlapped with the MIMIC blend. MIMIC blend activated relatively larger area as that stimulated by every single chemical. All tested odorants activated one single specific region in common (Fig. 3a, region of interest 01 [ROI01], red). No putative labelled line coding was observed since every standard chemical led to activity in discrete regions corresponding to specific glomeruli in the AL of *M. domestica* females. Among, ROI02 (purple) was activated by linoleic acid (L) only; ROI03 (yellow) was activated by both ethyl linoleate (EL) and methyl linoleate (ML); ROI04 (Green) was activated by EL and ethyl palmitate (EP); ROI05 (Blue) was activated by ML, L, and EL.

The maximum intensity of activity was assessed by *ANOVA* among all odorant samples at every ROI region (Fig. 3b). As for ROI01, FWB, EL and L evoked significantly higher activities compared with paraffin oil (PO) and EP treatments (F_6,28_ = 3.14, P = 0.018). For ROI02, all five treatments were significantly higher than EP and FWB elicited significantly higher activity than PO and EP (F_6,28_ = 4.09, P = 0.005). FWB, MM (Mimicked blend), EL, L and ML activated significantly higher responses than PO and EP for ROI03 (F_6,28_ = 15.57, P < 0.0001). EL evoked the highest activity in ROI04 and this was significantly higher than PO, FWB, and ML (F_6,28_ = 6.16, P < 0.0001). FWB, L and ML stimulated significantly higher reactions compared with EP and PO in ROI05 (F_6,28_ = 8.095, P < 0.0001). Within all tested ROIs, MM always stayed at a relatively higher level. Spatio-temporal patterns of neuronal activity were presented as curves for every ROI and peak activity was observed during frame 18 to 22 in all cases (Fig. 3c), and all tested chemicals presented higher intensities compared with PO control.

### Cage tests and field demonstration

**Figure 4:**
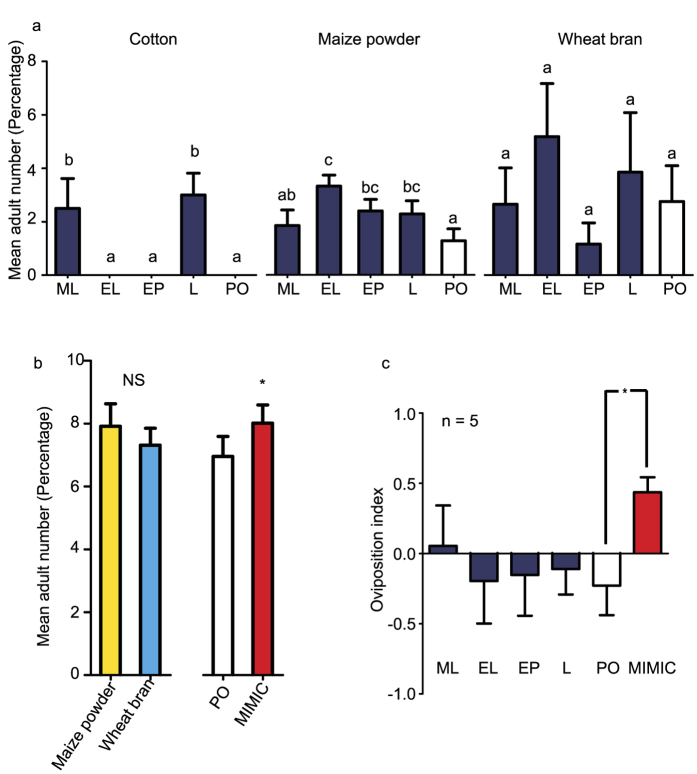
(**a**) Multiple-choice tests with females *M. domestica* on cotton, maize powder, and wheat bran. Paraffin oil was used as control. Error bars indicate ± s. e. m. (EL: ethyl linoleate, EP: ethyl palmitate, L: linoleic acid, ML: methyl linoleate, PO: paraffin oil control; *One-way ANOVA*, cotton: F_4, 45_ = 6.97, P < 0.001, maize powder: F_4, 145_ = 4.81, P = 0.001, wheat bran: F_4, 70_ = 0.86, P = 0.492, *Tukey HSD* multiple comparison test, similar letters above bars indicate no significant differences at 0.05 level between chemical treatments). **(b**) Two-choice tests for females *M. domestica* on two substrates. Paraffin oil was used as a control. Asterisk indicates significant difference between the control and MIMIC odorant treatment. (*Two-way ANOVA*, F_1, 105_ = 4.644, P = 0.034). Error bars indicate ± s. e. m. (**c**) Oviposition index in the field trial in Mali for the five compounds on chicken manure. The asterisk indicates a significant difference between the control and MIMIC odor (*Independent samples t test*, t = 0.023). Error bars indicate ± s. e. m.

Cotton fibers and maize powder were considered as non-substrate and non-preferred substrate for rearing *M. domestica* larvae respectively. Yet by adding chemical compounds to these substrates, the attractiveness of these substrates for oviposition was significantly increased. When cotton was used as a substrate, ML and L showed a highly significant increase in attractiveness towards female house flies (Fig. 4a, *ANOVA*, F_4, 45_ = 6.97, P < 0.001). EP, L and EL all increased the attractiveness of maize powder as compared with paraffin oil control (*ANOVA*, F_4, 145_ = 4.81, P = 0.001). Interestingly, no change in attractiveness was observed when attractants were placed on wheat bran (*ANOVA*, F_4, 70_ = 0.86, P = 0.492), a preferred substrate for *M. domestica*. MIMIC odor blend was significantly more attractive to females compared with the paraffin oil control (Fig. 4b, *two-way ANOVA*, F_1, 105_ = 4.644, P = 0.034), while maize powder or wheat bran as substrates did not affect the attractiveness of the chemicals (*Two-way ANOVA*, F_1, 105_ = 0.056, P = 0.813). A heat map^21^ was developed to show that a combination of wheat bran supplemented with the MIMIC odor blend would significantly increase attractiveness towards house fly adults (Supplementary Fig. S4, *two-way ANOVA*, F_5, 478_ = 3.835, P = 0.002, F_2, 478_ = 13.472, P < 0.001). The MIMIC fermented wheat bran odors did not stimulate an increased egg yield from female fly adults as compared to fresh wheat bran (Supplementary Fig. S5, independent samples *Mann-Whitney U test*, t = 0.2).

In the field trial in Mali, significantly more eggs were laid on chicken manure complemented with the MIMIC odor blend as compared to chicken manure alone (Fig. 4c, *Independent samples t test*, t = 0.023). However, single chemical compounds did not influence egg numbers laid in the substrates (*One-way ANOVA* and *Dunnett* multiple comparison, F_5, 24_ = 1.07, P = 0.402).

## Discussion

Wheat bran is considered as a nutritionally suitable substrate for rearing *M. domestica* larvae and it is widely used in laboratory rearing^22^. A previous study has confirmed that fermented wheat bran plus house fly ovaries can increase the number of eggs laid by gravid female house flies^15^. Our Y-tube olfactometer test not only further supported this result, but also revealed that both fermented wheat bran and rearing residue volatiles may attract female house flies when used alone. Since males were not significantly attracted by these odors, it implies that these may be involved in female behaviours such as oviposition site location and egg laying^23^.

The GC-EAD (electroantennographic detection combined with gas chromatography) method successfully identified ethyl palmitate, ethyl linoleate, methyl linoleate and linoleic acid from fermented wheat bran volatiles. In addition, decane, 2-butyl-1-octanol, nonadecane and squalene were identified from rearing residue volatiles. All compounds were found to elicit an EAG response in female, but not male, antennae. The chemical fingerprints of sampled blends revealed dramatic changes in volatiles after rearing maggots with fermented wheat bran. However, the attractiveness of rearing residues to females might relate to behaviours other than oviposition because the rearing residue is not suitable for rearing maggots. The behavioural bioassays further confirmed that the chemicals identified increased both substrate attractiveness and oviposition. However, these odor components did not improve the attractiveness of wheat bran towards female flies. Mean counts of being attracted individuals within 12 h would better reflect treatment effects in a more balanced way without increasing unnecessary variances especially when certain individuals would always prefer one of the treatments. As it has been demonstrated that gravid female house flies would be attracted by fermented wheat bran for oviposition^15^, we suggest that these chemicals might be unique and critical for house flies to locate their spawning material. Although we have found no evidence to suggest that mating effects influence substrate recognition in house flies as we tested only gravid females in this study, further investigation may enable other semiochemicals to be identified from substrate volatile chemical fingerprints. The egg laying behaviour of female house flies is influenced by various factors^9^, and olfaction is probably the main one^23^. Similar observations were observed in other Diptera such as the well-studied vinegar fly *Drosophila melanogaster*^24^.

Esters could be involved in oviposition because they represented approximately 75% of the bioactive compounds in fermented wheat bran odor. Esters have been reported as key chemicals, which function as fruitless gene trigger compounds and also mediate copulation and attraction behaviours between male and female *D. melanogaster*^25^. The chemicals reported included methyl palmitate^25^, which is a very similar chemical compound to ethyl palmitate found in this study. Dweck *et al*.^25^ showed, using the two-photon optical imaging method, that compounds have a ‘labeled line’^26^ neuronal pathway both in the antennal lobe and higher brain center. Together with 11-cis-vaccenyl acetate (cVA), the previously known pheromone of *D. melanogaster,* they play a key role in mating behaviour^25^. The chemicals that were identified from fermented wheat bran odor have also revealed their influence to house fly on attractiveness and egg laying. The neuronal pathway for these chemicals might be sufficiently conserved for mediating mating related behaviours, such as copulation and oviposition.

The spatial pattern in antennal lobes could reflect functional effects of a chemical odorant received by insect antennae^27^. Moreover, chemicals sharing the same evoked area in the antennal lobe are likely to be correlated with the same behaviour output^28^. Tested blend chemical MM overlapped in ROIs with FWB extracts and involved the same olfactory receptors (ORs) and olfactory sensory neurons (OSNs) strongly suggesting similar functions for these two samples^29^. Calcium imaging in AL of female house flies also revealed that they detect tested esters with distinct OSN populations which led to discrete activation patterns. Moreover, a conserved region in the AL (ROI01) elicited by all tested samples implied that one common feature, such as attractiveness might be shared by every single chemical compound. Different discrete regions of ROI04 for EP and ROI05 for ML, L, EL suggested that different carbon bone (16 C and 18 C) specific OSN populations on the house fly peripheral olfaction system. For MM sample, AL activation patterns stimulated by mixtures of 4 single components were not purely additive of every compound’s pattern respectively, which, implied that a possible mixture interaction existed at OSN and AL input levels^28^. This result also coincided with the EAG test which showed that when the four chemicals were mixed, they stimulated significantly higher electrophysiological responses as compared to each individual chemical. Furthermore, indoor bioassays and field trials have observed similar results showing that a blend of mimicked compounds performs significantly better than single chemicals in terms of attractiveness and oviposition acceleration.

Since odorant preference plasticity has been observed in *M. domestica* (Kelling *et al*.^30^ and personal observations conducted in Huazhong Agricultural University), chemical cues might have different coding and profiling for different strains that were reared under various substrate odorant backgrounds – in a similar way to the influence of a host plant on olfaction of house fly^31^,^32^,^33^. We selected attractants from fermented wheat bran based on the responses of a strain reared on the same substrate. The laboratory colony showed a strong and precise selectiveness towards fermented wheat bran chemicals (as in the Y-tube and GC-EAD experiments). While other strains, as used in cage bioassays, EAG and the field trial performed relatively poorly with the same chemical compounds, significant differences in attractiveness could be observed when these chemicals were applied to different substrates. Thus, we believe that those chemicals selected from fermented wheat bran could still be applied to the mass rearing of different house fly strains. Nevertheless, the variation between strains in their attractiveness towards fermented wheat bran chemicals needs further investigation.

Applications of the artificial mimicked wheat bran volatiles are potentially numerous^17^,^34^,^35^. *Musca domestica* is presently being domesticated in many parts of the world for animal feed but ensuring sustainable egg production is critical and results obtained so far suggest that egg production rates are much lower than the potential oviposition rate of house flies^4^,^7^,^9^. The chemical attractants identified herein could be used to enhance oviposition in mass rearing systems. Furthermore, as wheat bran itself can be considered costly, much cheaper substrates could be used if enriched with these chemicals. In West Africa, a procedure for rearing house flies has been developed based on the exposure of substrates to natural house fly oviposition^5^. Usually, animal manure is used as the substrate, often enriched with blood, fish offal or other animal waste. Chemical attractants could also be used in such systems, provided that they can be produced cheaply. Furthermore, by using attractants, cheaper, and potentially cleaner substrates could be used, such as fermented grass. Finally, *M. domestica* is also considered a pest in many environments and new management methods are needed^3^. The attractants found in this study could be used without the sex pheromone (*Z*)-9-tricosene, moreover an artificial mimicked blend of ethyl palmitate, ethyl linoleate, methyl linoleate and linoleic acid could be used to develop novel trapping lures for house fly control. However, more research is needed to optimize the use of such chemicals to enhance egg production in larvae production systems or in fly management.

## Methods

### Insects and substrates

For chemical screening experiments involving behavioural and electrophysiological studies, two laboratory colonies of *M. domestica* were mixed: a colony from Guangdong Entomological Institute, Guangdong Province, China and another from Huazhong Agricultural University, Wuhan Province, China. The two laboratory colonies had been reared on fermented wheat bran for 5 years and over 30 years respectively, with regular addition of wild specimens for rejuvenation.

Experiments to assess the effect of the volatiles identified, including dose response electroantennography and laboratory bioassays, used a mass produced strain of *M. domestica* purchased from Hefei Dayuan biology technical company, Anhui Province, China. The colony had been reared on a substrate mixture of fresh wheat bran, milk powder and fish meal for 11 years with regular rejuvenation.

In all laboratory tests, pupae were placed into containers for emergence at 25 °C, 70% RH and a photoperiod of 16:8 h L:D. Newly emerged adults were provided with raw chicken eggs for 3 days to accelerate the development of the ovaries in female flies before being used in cage assays. Gravid females and mated males were used in all cage tests. The wheat bran used in the experiments was the same as the one used in the lab rearing, which was purchased from local grain processing facilities in Guangzhou.

### Volatile odor sampling

The head space absorption method was used for collecting volatile blends from substrates, following previous protocols^36^,^37^. Odor compounds were collected at 25 °C using 0.3 g of super-Q absorbent (80/100, Alltech, Deerfield, IL, USA) for 24 h at 50 ml/min air flow rate and eluted with 1.5 ml hexane solvent. Ten grams (dry weight) of substrates used for head space absorption included: fresh wet wheat bran (60% water, w/w); fermented wheat bran (fresh wheat bran moistened with water at 60% w/w and fermented in a climate chamber at 30 °C for 48 h); residues obtained from larval rearing after consumption of fermented wheat bran; live third instar maggots. Volatile samples were not concentrated and were kept at −20 °C before being used in further tests.

### Y- olfactometer test

The attractiveness of volatile blends from substrates was tested with 1–3 day old adults using a Y-tube olfactometer (see Supplementary Fig. S6 for detailed device description and protocol). Pairwise comparisons were conducted with the volatiles collected from fresh wheat bran, fermented wheat bran and rearing substrate residue, Hexane was used as a control. To eliminate the potential influence of unseparated maggots in the rearing residue comparisons between the solvent and volatiles from larvae were also made. The choice made within three minutes was recorded and a total of at least 100 females and 50 males were tested with every single compound combination. All tests were conducted at room temperature, i.e. 25 ± 2 °C, with constant purified and moistened air flow at a rate of 0.5 l/min, and odorant compounds were switched between the two arms every 5th test. *Chi-square tests* were used to compare the attractiveness of each pair of volatile sources.

### Gas chromatography combined with mass spectrum (GC-MS)

GC-MS analysis was used to identify the chemical compounds for each of the volatile mixtures. An Agilent 5973N (Santa Clara, CA, USA) mass selective detector coupled with an Agilent 6890N network GC system equipped with a quartz capillary column (HP-5, 30 m × 0.25 mm × 0.25 μm; J&W Scientific, Palo Alto, CA, USA) was used. Injections were made in splitless mode with an injection port temperature of 280 °C. The oven temperature was initially held at 40 °C, increasing to 70 °C at 3 °C/min and then at 10 °C/min to 220 °C and held at 220 °C for 15 min. Helium was used as the carrier gas at 1 ml/min of constant flow rate. Candidate bioactive compounds were identified by crosschecking with mass spectrum fragment database and final confirmed with standard chemical spectrum patterns. The concentration of total chemical blends was calculated using a standard chemical curve developed from gradient GC-MS spectrums.

### Electroantennogram (EAG) combined with gas chromatography (GC-EAD)

GC-EAD was used for the identification of bioactive compounds in female flies. Antennae were processed following standard procedures by cutting both extremes of flagella and immediately mounted with two glass capillary Ag/AgCl electrodes containing Kaissling saline (glucose 354 mmol/l, KCl 6.4 mmol/l, KH_2_PO_4_ 20 mmol/l, MgCl_2_ 12 mmol/l, CaCl_2_ 1 mmol/l, NaCl 12 mmol/l, KOH 9.6 mmol/l, pH 6.5)^36^,^37^ and an identical GC column was used under the same temperature program as GC-MS with detector at 230 °C. Electroantennogram signals and flame ionization detector (FID) responses from the GC were recorded simultaneously. Bioactive chemicals were identified by crosschecking with GC-MS data. Both sexes of house fly adults were tested with headspace samples from wheat bran, fermented wheat bran and rearing residue.

Dose response curves were produced for standard chemical compounds (Purchased from Sigma, methyl linoleate (ML): >99% GC, TCI TOKYO Chemical Industry co. LTD.; ethyl linoleate (EL): >70%, TCI Shanghai branch, China; ethyl palmitate (EP): >95%, TCI Shanghai branch, China; linoleic acid (L): 60–74%, Ourchem, Shanghai, China; paraffin oil (PO): Chemical pure, Yong Da Chemical, Tianjin, China) using the EAG method on female house fly antennae. Ten antennae were used as replicates for each chemical at a single dose. Paraffin oil was used as the carrier solvent and the blank control. Five different concentrations were used in the experiments, i.e. 100 mg/ml, 10 mg/ml, 1 mg/ml, 0.1 mg/ml and 0.01 mg/ml for the four standard chemicals respectively. The EAG response was calculated using a standard calculation formula^36^ and then used for comparison among doses.

### Optical imaging of the antennal lobes (AL)

The calcium imaging method was adopted from Xu *et al*.^38^. A female house fly adult was mounted in an artificial block^39^ and vertex was sealed with two component silicon to let antennae open for odorants. After dissecting and exposing the brain, the antennal lobe was stained by a calcium-sensitive dye, CaGR-2-AM (Molecular Probes, Eugene, OR, USA) for one hour at 13 °C and then thoroughly rinsed with Ringer solution. For imaging we used a Till Photonics imaging system (Martinsried, Germany) equipped with a CCD camera (Retiga 2000R, QIMAGING) connected to an upright microscope (Olympus BX51WI). The antennal lobe was illuminated at 475 nm and odorant stimulation started at frame 13 and lasted 500 ms in the recording sequence of 40 frames. False color images were collected and relative changes in fluorescence (ΔF/F) were calculated and analyzed according to calcium changes. Four of the single chemical compounds plus one mixture MIMIC odorant were tested at a dose of 100 μg. Fermented wheat bran extraction and paraffin oil were used as positive and blank controls respectively.

### Choice tests in cage

Cage tests were conducted following a previously published protocol^15^. A two factorial design to test possible influences of both substrates and chemical samples was carried out. Standard chemicals were prepared as paraffin oil solutions at a concentration of 1 μg/μl. Four chemicals and paraffin oil as the control were provided simultaneously to female house flies. Gray rubber septa (The West Company, Phoenixville, PA) loaded with 100 μl of the test solution were used in the experiments. All chemicals were used at a dose of 100 μg per septum. Septa were put into 9 cm petri dishes together with substrates and all dishes were placed in cages (1.2 m × 1.2 m × 1.2 m) for attracting 20 couples of adult flies. Three different substrates: (cotton, maize powder and wheat bran at 60% water w/w and 10 g dry weight) were used with all five samples tested at once. The attractiveness was quantified by counting the number of individuals in the dishes once every hour and the experiment lasted for 12 h, and then mean numbers within 12 h were calculated and used for statistical analysis. Seven replicates were made for each type of substrate.

In another experiment, a volatile blend that mimicked the mixture in the fermented wheat bran (MIMIC) was prepared by mixing the four chemicals together, in the correct proportion (EP: EL: ML: L = 10: 24: 6: 0.2, with a concentration of 1 μg/μl in total). The MIMIC solution (100 μl) was dropped into gray rubber septa as the odor source, i.e. the equivalent dosage of odor blends was 100 μg. A two-choice test was carried out between the odor blend and the solvent control. Both maize powder and wheat bran were tested in two separate trials. Each substrate was replicated seven times. The number of flies recorded at each time point was transformed into percentages and a *one-way ANOVA* with *Duncan’s* multiple comparison was conducted to analyze differences among chemicals. A further oviposition test was carried out comparing MIMIC odor and paraffin oil control in wet wheat bran. The same protocols were followed as for the previous attractiveness test. When oviposition ceased after 3 to 6 h, eggs were collected from each dish and counted (∼15,000 eggs/g)^16^, and the number of eggs was compared between the two odors.

### Field demonstration

A field demonstration trial was carried out at a local house fly larvae production facility in Bamako, Mali (12,6550 N; 7,9268 W). In this production system, larvae are obtained by exposing substrates to natural oviposition^6^ (see Charlton *et al*.^6^ for a description of the rearing system). Wet chicken manure, a commonly used rearing substrate, was used as the experimental substrate. Five treatments of chemical compounds were mixed into solutions at the same proportion as in laboratory tests: ML, EL, EP, L and MIMIC odor mixture. Paraffin oil was used as the control. Wet chicken manure were placed in 9 cm petri-dishes and 50 μl of a solution was placed on the substrate. The dishes were randomly distributed in an open house fly rearing site facility and after 5 h they were covered with their lid. The number of larvae was counted two days later. Larvae numbers were transferred into oviposition indexes using the formula: (O − C)/(O + C) (O = larvae numbers from sample, C = larvae numbers from control) within one replicate. A total of five replicates were made. *One-way ANOVA* and *Dunnett* multiple comparison tests were used to identify the influence of the tested chemical on oviposition.

### Statistics

All statistics were carried out using IBM SPSS Statistics 22.0.0 (SPSS Inc., Chicago, IL, USA). Bar charts were developed by Prism 5 for Windows ver. 5.01 (GraphPad software Inc., San Diego, CA, USA). A heat map chart was developed by SPSS together with Microsoft Excel 2013 and edited by Microsoft Powerpoint 2013 (Microsoft, San Francisco, CA, USA). Calcium imaging graphics were processed with MATLAB 7.8.0.347 (The MathWorks, Inc., Natick, MA, USA).

## Additional Information

**How to cite this article**: Tang, R. *et al*. Identification and testing of oviposition attractant chemical compounds for *Musca domestica.* *Sci. Rep.* **6**, 33017; doi: 10.1038/srep33017 (2016).

## Supplementary Material

Supplementary Information
