# Supplementary material for: Identification and testing of oviposition attractant chemical compounds for Musca domestica
Source: Sci Rep. 2016 Sep 26;6:33017. doi: 10.1038/srep33017 (PMC5036095; doi:10.1038/srep33017)
Supplement: Supplementary Information [file srep33017-s1.doc]

Identification and testing of oviposition attractant chemical compounds for *Musca domestica*

TANG Rui^*^, ZHANG Feng, KONE N’Golopé, CHEN Jing-Hua, ZHU Fen, HAN Ri-Chou, LEI Chao-Liang, KENIS Marc, HUANG Ling-Qiao, WANG Chen-Zhu

**Supplementary materials**

**Supplementary Table 1.** Chemical compounds identified within four different head space absorbed samples by GC-MS analysis.

| **Samples** | **Chemicals** | **Retention time (min)** |
| --- | --- | --- |
| Fresh Wheat Bran  10 compounds | 1-Pentanol  Hexanal  1-Hexanol  2-pentyl-Furan  (*E*)-4-Nonenal  (*E*)-2-Nonenal  2,4-Decadienal  4,4,6-Trimethyl-cyclohex-2-en-1-ol  n-Hexadecanoic acid  9-12-Octadecadienoic acid (*Z*,*Z*)- | 4.343  4.979  6.861  11.215  13.943  15.033  17.563  18.326  25.647  27.891 |
| Larvae  16 compounds | sec-Butyl fluoroformate,  3-Hexanol  4-methyl-2-Pentanol  2,4-dimethyl-Hexane  Ethylbenzene  1-chloro-Hexane  Undecane  O-decyl-Hydroxylamine  2,6,10,14-tetramethyl-Heptadecane  Nonadecane  1-(2-isopropyl-5-methylcyclohexyloxy)-3-(1-piperidyl)-Propan-2-ol  Phthalic acid butyl undecyl ester  1-(ethenyloxy)-Octadecane  (*Z*)-7-Hexadecenal  Dibutyl phthalate  n-Nonadecanol-1 | 4.407  4.944  5.043  5.538  6.634  6.891  13.081  21.579  22.791  23.91  24.272  24.61  24.837  25.111  25.583  26.008 |
| Fermented Wheat Bran  18 compounds | Butanoic acid  Butanoic acid, ethyl ester  Acetic acid, butyl ester  1-Hexanol  Butanoic acid, butyl ester  Propanoic acid, 2-hydroxy-, butyl ester  2-Methoxy-phenol  2(3H)-Furanone, dihydro-5-pentyl-  Ethyl-π(4-hydroxy-3-methoxy-phenyl)-propionate  Ethyl palmitate*  Ethyl linoleate*  (*E*)-9-Octadecenoic acid ethyl ester  Hexadecanoic acid, butyl ester  Methyl linoleate*  Linoleic acid*  17-Octadecynoic acid  2-Methyl-*Z*,*Z*-3,13-octadecadienol  Z-10-Tetradecen-1-ol acetate | 4.676  4.792  5.008  5.323  6.855  11.373  11.746  13.856  18.618  23.391  25.833  28.194  28.287  28.585  32.49  33.22  34.36  35.78  37.65 |
| Rearing Residue  25 compounds | 2-Nonanone  1-Ethylbutyl-hydroperoxide  2-Hexanol  Pyrazine, trimethyl-  Decane, 2,9-dimethyl-  Pyrazine, tetramethyl-  Decane*  2,6,6-Trimethyl-2-cyclohexnene-1,4-dione  2-Methyl-undecane  2,6-Dimethyl-heptadecane  Octanal  9-Octadecenal  Dodecane, 2,6,11-trimethyl-  2-Butyl-1-octanol*  O-decyl-hydroxylamine  Tricyclo[5.2.1.0(1,5)]decane-8,9-diol  Oxirane, tetradecyl-  Didodecyl phthalate  Nonadecane*  Dodecane, 2,6,10-trimethyl-  2-Hexyl-1-octanol  Octadecane, 1-(ethenyloxy)-  Oxirane, tetradecyl-  Dodecanal  Squalene* | 4.821  4.944  5.043  11.542  13.08  13.768  14.106  14.986  17.318  20.331  21.27  22.43  22.861  23.333  23.426  24.102  24.312  24.627  24.831  25.105  25.565  25.764  25.903  28.625  29.167 |

* Asterisks indicate chemicals that stimulated bioactivities in electric physiological experiments.

**Supplementary Figure 1.** **Results of two-choice tests by *Musca domestica* males in Y-olfactometers** (X-axis digits indicate counts of female houseflies, and adults without chosen action were not included in the figure. FWB: fermented wheat bran, WB: wheat bran, RES: residue, CK: solvent control, LAR: larvae odor).

**Supplementary Figure 2. GC-EAD results for *M. domestica* males and females** (a: female adult response to rearing residue odorant blend; b: male adult response to rearing residue odorant blend; c: male adult response to fermented wheat bran odorant blend; d: male adult response to fresh wheat bran odorant blend; e: female adult response to fresh wheat bran odorant blend. 1: Decane, 2: 2-Butyl-1-octanol, 3: Nonadecane, 4: Squalene).

**Supplementary Figure 3. EAG intensity comparison among four standard chemicals and MIMIC odorant blends at a dose of 100ug for male *M. domestica* antennae.** (Error bars indicate + s.e.m. EL: ethyl linoleate, EP: ethyl palmitate, L: linoleic acid, ML: methyl linoleate, MM: mimic blend; *ANOVA*, *Tukey HSD*, F_4, 61_=2.45, P=0.055).

**Supplementary Figure 4. Heat map^1^ showing attractiveness test using multiple chemicals on different substrates.** Asterisks indicate significant differences among substrates and chemical odors (*F_5, 478_=3.835, P=0.002, ** F_2, 478_=13.472, P<0.001, *two-way ANOVA*, *Tukey HSD* multiple comparison test).

**Supplementary Figure 5.** **Oviposition lab test using MIMIC odor blend versus paraffin oil control in wheat bran as substrate.** Error bars indicate + s.e.m. No significant difference was observed in the number of eggs laid between MIMIC odor and paraffin oil control (*independent samples Mann-Whitney U test*, t=0.2).

**Supplementary Figure 6. Methodology for two choice tests using Y-tube olfactometer.** The system was adopted from reported works on *M. domestica*^2^. A glass made Y-tube was developed with the main arm at 50 cm and the two-choice arms at 20 cm in length. Two 10 cm long odor chambers were attached at both ends and the inner diameter was 3 cm. Odor chambers were removeable and each chamber was prepared affiliated with every treatment odorant source to avoid possible chemical pollutions among treatments. The odor chambers were sealed from outside with gauses for preventing flies from escaping (blue color). Air was firstly introduced via an air sampler (QC-1, Beijing Municipal Institute of Labour Protection, Beijing,PRC; section 1), and then air flow rates were synchronized by two flow meters (section 2). The air was purified by powdered activated carbon in section 3 and moistened with double-distilled water in section 4. And then prepared air was pumped into Y-tube with constant flow rate and individual flies were introduced from the other end of the tube for conducting choice action. The system was illuminated with four parallel fluorescent lamp (TLD 36W/54 DAYLIGHT, PHILIPS LIFEMAX) with 3 meters of vertical distance from overhead. The two chambers containing odorant sources (gray rubber septa loaded with sample solutions, The West Company, Phoenixville, PA) were altered every 5 tests to reduce possible environment influences.

References

1. Wilkinson, L. & Friendly, M. The history of the cluster heat map. *Am. Stat.* **63**, 179-184 (2009).

2. Haselton, A. T. *et al.* Repellency of α-pinene against the house fly, *Musca domestica*. *Phytochemistry* **117**, 469-475 (2015).
